# Ensembl comparative genomics resources

**DOI:** 10.1093/database/bav096

**Published:** 2016-02-20

**Authors:** Javier Herrero, Matthieu Muffato, Kathryn Beal, Stephen Fitzgerald, Leo Gordon, Miguel Pignatelli, Albert J. Vilella, Stephen M. J. Searle, Ridwan Amode, Simon Brent, William Spooner, Eugene Kulesha, Andrew Yates, Paul Flicek

**Affiliations:** ^1^European Molecular Biology Laboratory, European Bioinformatics Institute, Wellcome Trust Genome Campus, Hinxton CB10 1SD,; ^2^Bill Lyons Informatics Centre, UCL Cancer Institute, University College London, London WC1E 6DD,; ^3^Wellcome Trust Sanger Institute, Wellcome Trust Genome Campus, Hinxton CB10 1SA,; ^4^Eagle Genomics Ltd., Babraham Research Campus, Cambridge, CB22 3AT, UK, and; ^5^Cold Spring Harbor Laboratory, 1 Bungtown Road, Cold Spring Harbor, NY 11724, USA

## Abstract

Evolution provides the unifying framework with which to understand biology. The coherent investigation of genic and genomic data often requires comparative genomics analyses based on whole-genome alignments, sets of homologous genes and other relevant datasets in order to evaluate and answer evolutionary-related questions. However, the complexity and computational requirements of producing such data are substantial: this has led to only a small number of reference resources that are used for most comparative analyses. The Ensembl comparative genomics resources are one such reference set that facilitates comprehensive and reproducible analysis of chordate genome data. Ensembl computes pairwise and multiple whole-genome alignments from which large-scale synteny, per-base conservation scores and constrained elements are obtained. Gene alignments are used to define Ensembl Protein Families, GeneTrees and homologies for both protein-coding and non-coding RNA genes. These resources are updated frequently and have a consistent informatics infrastructure and data presentation across all supported species. Specialized web-based visualizations are also available including synteny displays, collapsible gene tree plots, a gene family locator and different alignment views. The Ensembl comparative genomics infrastructure is extensively reused for the analysis of non-vertebrate species by other projects including Ensembl Genomes and Gramene and much of the information here is relevant to these projects. The consistency of the annotation across species and the focus on vertebrates makes Ensembl an ideal system to perform and support vertebrate comparative genomic analyses. We use robust software and pipelines to produce reference comparative data and make it freely available.

**Database URL:** http://www.ensembl.org.

## Introduction

The number of publicly available chordate genomes has been increasing at a fast pace since the publication of the human genome sequence (1, 2) and is expected to increase further in the coming years due to continuous advances in sequencing technologies. One of the first common analyses when sequencing a new genome is to compare it with previously analysed genomes. In fact, comparative analysis is such an important tool to better characterize genomes that a set of 29 mammalian genomes, including 22 specifically sequenced for the project, were analysed together as a means to understand the human genome (3).

Comparative genomics analyses can focus on the similarity and differences between the annotation or between the sequence of two or more genomes. Pairwise and multiple whole-genome alignments are used to compare genome sequences. Several software packages (4–6) exist to detect conserved regions from a multiple alignment. Pairs of genes can be annotated as orthologues or paralogues (7). Orthologues represent genes related by a speciation event while paralogues are genes related by a duplication event. Despite recent concerns on the orthology conjecture (8), orthologues tend to be more similar in function than paralogues (9) and are widely used in gene annotation (10, 11).

Ensembl provides comparative analyses at both the genomic and genic levels. Genome sequences are compared using pairwise and multiple whole-genome alignments and based on these alignments, synteny, sequence conservation scores and constrained elements are determined. Gene homology relationships are represented by GeneTrees (12), while Ensembl Protein Families serve as a powerful way to find sequence similarities between protein sequences in Ensembl and in UniProt (13). All these multi-species data resources are stored centrally in the Ensembl ‘Compara’ database.

Other comparative resources are available. These include the UCSC genome browser (14), which provides several sets of whole-genome pairwise and multiple alignments, as well as conservation data; and the VISTA Browser (15), which provides additional sets of multiple alignments (16) that can be viewed on either the VISTA browser itself or as an additional track on the UCSC genome browser. Other databases provide alternative phylogenetic trees (17–20) or sets of orthologues, including those provided by OMA (21), COGs (22) and HomoloGene (23). Notably, other projects including Ensembl Genomes (24) and Gramene (25) provide comparative genomics data based on the infrastructure and pipelines described here.

We have previously described our algorithm for producing protein-coding orthology and paralogy annotations (12) as well as the algorithms used to create our whole genome multiple alignments (26, 27). Here, we provide a comprehensive overview of the suite of Ensembl comparative genomics resources, the detailed methods used to produce them and the tools available to access and use the data. The descriptions here are complementary to the brief updates provided in annual Ensembl publications, which are more focused on short highlights across the breadth of Ensembl. With approximately five updates per year, the Ensembl Compara database is the most comprehensive and up-to-date comparative genomics resource for vertebrate genomes. The data are accessible through the web interfaces, such as the public MySQL server and the Perl and REST APIs. Most of these are also downloadable from our FTP server.

## Methods

Ensembl provides comprehensive evidence-based annotation of all genome sequences that it supports. Depending on the species-specific availability of protein sequences and gene expression data such as RNA-Seq, cDNA or ESTs, and the quality of the assembly for a particular species, different strategies are used to create the Ensembl gene set (http://www.ensembl.org/info/genome/genebuild/genome_annotation.html). Despite the differences between these approaches, the end result is gene annotation across all species that is relatively consistent and therefore suitable for comparative analysis.

Ensembl data are updated regularly: unless stated otherwise, this article describes the characteristics of Ensembl comparative genomic resources for release 80 (May 2015; http://e80.ensembl.org). Specific details for the most current release are available at http://www.ensembl.org.

A new and fully updated Ensembl Compara database is created with every Ensembl release. We use the eHive (28) workflow system to manage all of the computational pipelines. The set of alignments to be updated in each release is generally large enough to require the execution of multiple eHive-managed pipelines running in parallel. The results from each of these analyses feed into a separate production database instance, which captures the results of one pipeline. Whenever the component genome sequences in a given alignment have not changed from one release to the other, we reuse alignments from the previous release to save computing time. Finally, we merge previous alignments and new data into a single Ensembl Compara database.

In order to ensure consistency between Ensembl releases, we use an internal master database. This is an additional instance of an Ensembl Compara database that holds all the entries of the species, genomic sequences and analyses. New species, analyses, etc. are added to the master database before starting the production pipelines, such that all the relevant internal IDs are consistent across databases.

For practical and algorithmic purposes, the data provided for a given species depend partly on the characteristics of its genome assembly. For example, depending on assembly contiguity and completeness some analyses require special consideration or are inapplicable. In the era of Sanger-based sequencing technology, genome assemblies resulting from at least 6 × sequence depth generally produced highly-contiguous, largely-complete genome assemblies often referred to as ‘high-coverage’, while ‘low-coverage’ sequencing resulted in more fragmented and incomplete assemblies (3). Although sequencing depth from high-throughput short read sequencing technology does not correlate with assembly quality in the same way and the absolute numbers have changed, as is common practice, we will use the terms ‘high-coverage’ and ‘low-coverage’ to refer to assembly contiguity and completeness.

### Whole-genome pairwise alignments

**Figure 1.**
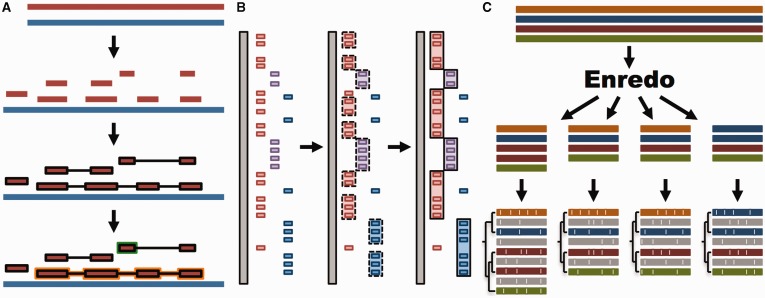
Whole genome analysis pipeline. (**A**) Pairwise alignments. A reference genome (blue) is aligned to another genome (red) with LASTZ. The raw alignments that are in the same order and orientation are grouped in chains (highlighted in black). On each region of the reference genome, the best chain is selected to single out the set of nets. A top-level net (orange) can include a nested net (green) in regions it does not cover. (**B**) Large-scale syntenies. LASTZ-net alignments are sorted on a reference genome (grey). The red, magenta and blue boxes represent alignments to different chromosomes in the other genome. For simplicity, we assume that they are in the same order and orientation. Contiguous collinear alignments are joined in a first-pass, forming a nascent syntenic block. In the second pass, the nascent blocks are joined and extended further to build macro-synteny blocks. (**C**) EPO multiple alignments. The sequences of all genomes are fed into Enredo to build sets of collinear blocks. These are aligned with Pecan and Ortheus resulting in an alignment with inferred ancestral sequences (in grey).

We use LASTZ (29) to build pairwise alignments. LASTZ represents a new implementation of the previous BLASTZ (30) algorithm and includes several improvements (29). We post-process the alignments such that runs of compatible alignments (in order and orientation) are joined in so-called ‘chains’ (31). Further, we refine the final list of chains by using the axtNet (31) software. Given a reference genome, axtNet returns the best chain in each region of the reference genome. These chains are termed ‘nets’. Some nets can be embedded in the internal gaps of longer nets, creating a nested structure. Figure 1A shows an overview of this process. The final nets represent a highly stringent set of alignments and are the ones accessible through the web interface. Until release 71, pairwise alignments across different clades (e.g. from eutherian mammals to birds or teleost fish) were built using translated BLAT (32). Starting from Ensembl release 72, all new alignments are built with LASTZ.

The human genome sequence is aligned to every vertebrate genome sequence in Ensembl. Additional pairwise alignments are provided for a few key species (mouse, dog, chicken, zebrafish and medaka). Other pairs of species may be aligned based on specific scientific interest or by community request and currently include pig-cow, pig-sheep, opossum-wallaby, stickleback-cod and others.

The nucleotide similarity matrix and specific LASTZ parameters we use depend on the pair of species aligned. In general, we use more stringent parameters when aligning two closely related species such as two primate genomes (Table 1). This is necessary as the sequences are highly similar and default parameters create too many spurious alignments. The actual parameters used for a given alignment are listed at http://www.ensembl.org/info/genome/compara/analyses.html.

**Table 1.** LASTZ alignment parameters

Parameter	Primates				Other			
Gap open penalty (O)	400				400			
Gap extend penalty (E)	30				30			
HSP threshold (K)	5000				3000			
Threshold for gapped extension (L)	5000				3000			
Threshold for alignments between gapped alignment blocks (H)	3000				2200			
Masking count (M)	10				–
Seed and transition value (T)	1				1			
Scoring matrix (Q)	**A**	**C**	**G**	**T**	**A**	**C**	**G**	**T**
	90	−330	−236	−356	91	−114	−31	−123
	−330	100	−318	−236	−114	100	−125	−31
	−236	−318	100	−330	−31	−125	100	−114
	−356	−236	−330	90	−123	−31	−114	91

### Large-scale syntenies

We define syntenic blocks from whole-genome pairwise alignments with the aim of producing an overview at the chromosome level of large-scale rearrangements or lack thereof. As shown in Figure 1B, we use a two-step approach. First, we group all consecutive pairwise alignment blocks that are in the same order and orientation. To be included in a group, blocks must not be separated by more than 200 kb in either genome. We then join the resulting groups into large-scale syntenic blocks, incorporating small-scale internal rearrangements between groups as long as they represent <3 Mb on either genome. These empirically derived thresholds work well for vertebrate genomes where most syntenic regions are >1 Mb and few exceed 100 Mb in length.

Since we derive the syntenic blocks from the whole- genome alignment, we only provide these for a selection of pairs of species. It is also worth noting that these syntenic blocks are not intended to show duplications in one species with respect to another. Duplications are presented in the Enredo-Pecan-Ortheus (EPO) whole-genome alignments (26, 27) which specifically support such features and are described below.

### Whole-genome multiple alignments

Our multiple alignments are built with Pecan (26), which uses a consistency-based approach to obtain high-quality alignments (33, 34). Essentially, Pecan improves the alignment between any two sequences (A and B) by using information from alignments to a third sequence (A-Xn and Xn-B). Pecan favours an alignment between A and B that is consistent with the A-Xn and B-Xn alignments. Pecan produces global alignments (i.e. it aligns the sequences from start to end) using a set of collinear sequences as input.

We define collinear regions using two different strategies. Mercator (35) builds sets of orthologous loci by looking at best reciprocal exon–exon alignments, i.e. an exon in one species being the most similar to another exon in the other species and vice versa. Using coding exons for this step gives us more sensitivity when comparing the genomes of distantly related species at a marginal cost of a bias towards protein-coding genes in the resulting orthology map.

We developed Enredo (26), which includes support for segmental duplications, to overcome some of the limitations of Mercator. In brief, Enredo can use any sequence, whether it is exonic or not, to build the collinear segments. These sequences, referred to as Genomic Point Anchors (GPAs), are mapped on the genomes and serve as the nodes of the Enredo graph. The edges in the graph are the genomic segments between the GPAs in the original assemblies. An edge with two or more genomic segments represents a collinear segment. Other graph transformations are allowed to manage missing or spurious GPAs, which result in longer and more meaningful segments. Because Enredo has no restriction in the content of the edges, the final collinear segments can contain any number of copies in every genome providing a natural way to incorporate the duplications into the alignment.

Ortheus (27) is an optional step in the multiple- alignment pipeline used to predict ancestral sequences by inferring the most probable collection of insertion and deletion events in the history of the sequences given its evolutionary model. The ancestral sequences can then be used to predict the age of each base of any extant species, and call ancestral alleles for its SNPs. For the specific case of high-coverage primate genomes, ancestral alleles are extracted from the EPO multiple alignments and are available on the FTP site as FASTA files. While Ortheus is a fully featured sequence aligner, it lacks many of the heuristics built into common sequence aligners. Thus, to run efficiently, Ortheus relies on an existing multi-sequence alignment (calculated by Pecan in our case). Although it has the capacity to review the input alignment, we do not allow Ortheus to change the original Pecan alignments as this would require additional computing time and the quality of the final alignments does not materially improve.

We combine all three programs to create the EPO multiple alignments for specific clades (Figure 1C). In Ensembl release 80, these include an 8-way primate alignment, a 17-way placental mammal alignment, a 4-way sauropsids alignment and a 5-way teleost fish alignment. Because of limitations in Enredo for building a reliable colinearity map among more distantly related species like human and chicken, we use a pipeline that combines Mercator and Pecan to generate a 23-way amniote alignment. All these alignments include high-coverage genomes only.

Aligning highly fragmented or low-coverage assemblies poses several technical problems in our analysis. For example, Enredo assumes that the genomic sequences are organized in chromosomes and low-coverage genomes are typically assembled in contigs only. We have designed a hybrid approach that first aligns the high-coverage genomes and then maps the remaining sequences to the multiple alignments. Thus, we benefit from the quality of the consistency-based multiple alignments while permitting the inclusion of many low-coverage genomes that could not otherwise be accommodated by the EPO pipeline. As a side effect, the scalability of the whole process is improved as the most computationally expensive step, building the consistency-based multiple alignments, is run with a smaller number of genomes.

**Figure 2.**
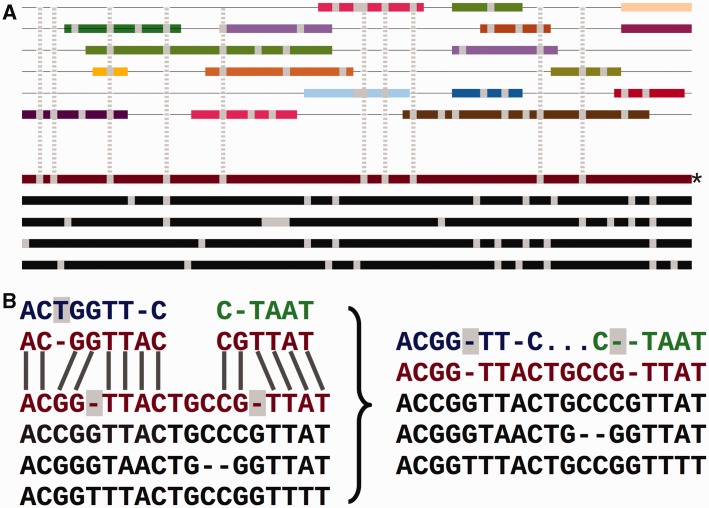
Adding the secondary set of species to an EPO alignment. (**A**) Overview of the process. The lower part of the panel represents the initial consistency-based multiple alignment, where the red line represents the human sequence. The upper part shows a mosaic structure for each secondary species. The grey vertical lines show the gaps added to the secondary genomes to accommodate them in the multiple alignment and how they match the deletions in the human sequence. (**B**) Detailed view on the removal of species-specific insertions and addition of gaps in a secondary genome. The left-hand side of the panel shows a segment of the multiple alignment and the matching pairwise alignments to a secondary genome. The right-hand side of the panel shows the resulting alignment. The highlighted blue T on the left-hand side is removed from the final multiple alignment. The deletions in the human lineage (also highlighted) are added in the secondary genome.

Figure 2A shows how the low-coverage genome sequences are mapped into the multiple alignment using pairwise alignments between the given genome and a reference species. The mapping results both in some positions being ignored and additional padding gaps being inserted into these genomes (Figure 2B). This process facilitates efficiently adding many genomes into the larger multiple alignment, while partially maintaining the quality of the consistency approach. We provide an all-placental mammalian EPO-LOW-COVERAGE alignment, using human as a reference species. A similar approach is used for teleost fish and sauropsids, using zebrafish and chicken, respectively, as the reference genomes for mapping the low- coverage genomes.

### Sequence conservation

Regions of evolutionary conservation in genome sequences can be estimated from a multiple alignment. We use GERP (4) to calculate a per-base conservation score, which represents how much a given column in the alignment is conserved across all the sequences. In a second pass, GERP uses a permutation test to define constrained elements—also known as conserved regions—as specific segments of the alignment that appear to be more conserved than expected by random chance. We store the conservation scores at different resolution levels for optimal database retrieval and display on genomic regions of various sizes. For example, the average conservation score is stored separately for each 10, 100 and 500 bp window.

**Figure 3.**
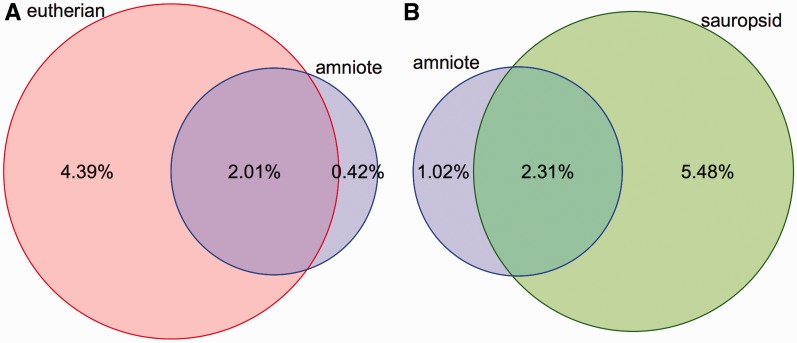
Coverage of constrained elements on the human and chicken genomes. (**A**) Overlap between the eutherian and amniote constrained elements on the human genome. The amniote elements cover a smaller portion of the genome because the 23-way amniote Mercator-Pecan alignment coverage is smaller and because elements that are conserved only in eutherian mammals might be missed when looking at all amniotes. (**B**) A similar plot for the chicken genome. Sauropsid-specific elements extracted from a 7-way sauropsid EPO alignment and the 23-way amniote Mercator-Pecan alignment are compared. In both cases, there is a fraction of the genome that is specifically detected as conserved when looking at all the amniotes. These regions are likely to be only mildly conserved and require the inclusion of more distant species to be detected.

Conservation scores and elements are provided for four sets of alignments: amniote Pecan, placental mammals EPO-LOW-COVERAGE, sauropsids EPO-LOW-COVERAGE and teleost fish EPO-LOW-COVERAGE. Conservation scores are not provided for the primate alignment set because the phylogenetic distance among the species is too short to be able to detect constrained elements. Conservation scores are also not provided for the other EPO alignments, which are a subset of the corresponding EPO-LOW-COVERAGE alignments and, thus, offer less information. In contrast, separately calculated conservation scores and constrained elements for birds, placental mammals and amniotes enable the detection of regions that are conserved in mammals only, in birds only or are conserved in all amniotes (Figure 3).

### GeneTrees and orthologies

**Figure 4.**
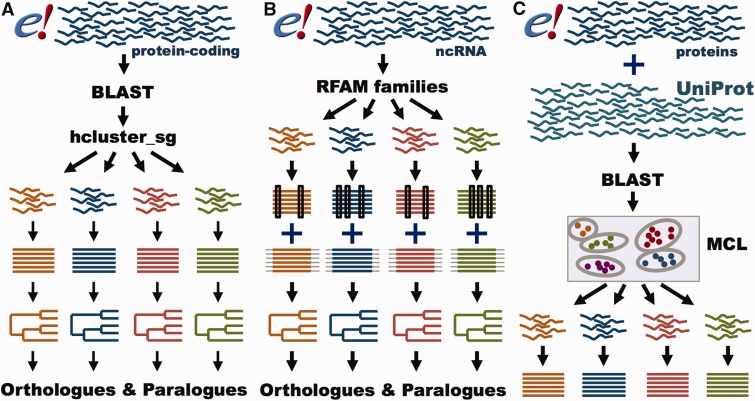
GeneTree and Ensembl Protein Family pipelines. (**A**) GeneTree pipeline for protein-coding genes. For each protein-coding gene in Ensembl, a representative protein is used. BLAST scores are provided to hcluster_sg for grouping the sequences into gene families. The proteins are aligned with MCoffee or MAFFT and a phylogenetic tree is built with TreeBeST. Finally, orthologues and paralogues are inferred from the tree. (**B**) GeneTree pipeline for ncRNA genes. Short ncRNA genes in Ensembl are grouped according to their RFAM classification. Both Infernal and PRANK alignments are used to build several phylogenetic trees that are merged into a final model with TreeBeST. Finally, orthologues and paralogues are inferred from the tree. (**C**) Ensembl Protein Family pipeline. All proteins in Ensembl and all metazoan proteins in UniProt are used. BLAST scores are fed into MCL to group the sequences by their similarity. The proteins are aligned with MAFFT.

GeneTrees are built with all protein-coding genes in Ensembl, as well as three non-chordate model species, *Caernobditis elegans*, *Drosophila melanogaster* and *Saccharomyces cereviciae*, using our previously published method (12). In short (Figure 4A), one representative protein from each gene is used in an all-vs-all approach using BLAST (36), followed by a Smith–Waterman alignment (37) to obtain the best alignment score for every pair of homologous proteins. We then use hcluster_sg (http://tree soft.svn.sourceforge.net/viewvc/treesoft/branches/lh3/hclus ter), a phylogeny-aware clustering algorithm, to collect the genes in groups (about 20 000 in release 80) based on the BLAST e-values. These groups are then aligned with M-Coffee (38) and the GeneTrees are inferred with TreeBeST (https://github.com/Ensembl/treebest). For large or complex groups, MAFFT (39) is used to create the alignment for TreeBeST. Methodological improvements compared with our previously published approach (12) include optimization of the clustering step we use to define sets of homologous proteins and the use of M-Coffee to build the multiple alignments. We have also worked extensively to improve orthologue and paralogue assignment in cases where genes are incompletely annotated, as described below. Information on the latest version of the pipeline is available at http://www.ensembl.org/info/genome/compara/homology_method.html.

Given a phylogenetic tree, it is straightforward to classify genes according to the classical definition of orthology and paralogy (7). However, in order to apply these rules, the nodes of the gene tree must be annotated as either duplication or speciation events. This is done with TreeBeST, which uses the species tree both to guide the phylogenetic reconstruction and also to reconcile the resulting gene tree with the species tree. The reconciliation marks a node as a speciation or duplication event by comparing the gene tree to the species tree. Nodes in the tree can be dated phylogenetically using the species underneath that particular node. Since every pair of orthologous or paralogous genes is related by a given node in the tree, we can provide an approximate time since the most recent common ancestor. The precision of the time estimation depends, of course, on the taxon sampling for the relevant part of the species tree.

Often, the data do not fit the model correctly. As a result, the gene tree does not match the species tree and additional duplication nodes are called in the reconciliation step (40). We use the duplication consistency score (12) to detect artefactual duplications. This score compares the number of species where this duplication is present with the total number of species under the node in question. A score of 0% means that the duplication event is not supported by any duplication in any extant species. We name these nodes dubious duplications or ambiguous nodes and consider them as speciation events for orthology extraction. This method has been shown to substantially improve the interpretation of the trees (12, 41).

The phylogenetic interpretation of duplication nodes with a low consistency score is ambiguous. These nodes should relate paralogous genes, but often the low consistency score reflects problems in either the input sequences or the inadequacy of the phylogenetic model used to infer the tree. When the consistency score is <25%, we tag as orthologues the resulting pairs of genes that don’t yet have an orthologue assignment. They are labelled as ‘not compliant’ to the gene tree (as well as the pairs originating from ambiguous nodes). We recommend that all the pairs of orthologues be considered unless a strict conformance to the gene-tree and the classical definition of orthology is required.

**Figure 5.**
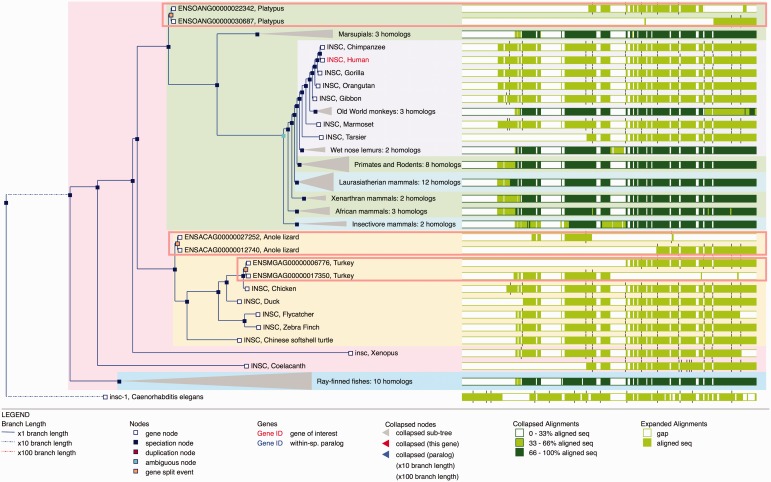
Gene Tree with split genes. GeneTree for the INSC gene in Ensembl release 80. The blue nodes in the tree represent speciation events and the light brown nodes are gene split events. The background color is used to show the different species clades (sauropsids, primates, teleost fish, etc.). Some nodes are collapsed (grey triangles) and show a summary of that sub-tree. The right part of the figure shows an overview of the alignment where the white areas correspond to gaps in the protein alignments. The three light brown rectangles highlight the three gene-split events in this family. The alignment overview for these genes clearly shows how the genes have been split.

A difficulty in homology assignment arises from genome annotation artefacts in areas where the genome assembly has not been correctly resolved. For example, when a contig is missing, inverted or appears in the wrong position, it might be impossible to construct a full-length gene model. In these cases, two or more partial genes may result from the gene annotation pipeline and they will incorrectly appear to be related by the presence of additional duplication nodes in the phylogenetic tree. We detect possible split genes within the multiple alignment by scanning for cases where sequences from putative split genes do not overlap (we actually allow for a small overlap to address misalignments and over-predictions of gene boundaries). When these cases are detected, we merge the component parts of the gene such that they appear together in the tree and rename these nodes as ‘gene split’ events (Figure 5).

Gene gains and losses in each GeneTree are calculated by starting from the number of gene copies in each species and using CAFE (42) to estimate how many genes existed in each lineage before a speciation event. In addition to the actual estimates, CAFE includes a statistical test to highlight the expansion or contraction events that are less likely to happen by chance. We also compute *dN*/*dS* values for pairs of orthologues that diverged within the last ∼100 million years, namely between pairs of mammalian, avian or tetraodontiforme genomes, using the codon-based model (43) implemented in PAML (44).

In addition to the GeneTrees for protein-coding genes, we have recently developed a method to create phylogenetic trees for short ncRNA genes (Figure 4B) (45). In brief, ncRNA genes are classified according to their Rfam (46) or miRBase (47) annotation. The ncRNA gene sequences are aligned with Infernal (48), while the flanking regions are aligned with PRANK (49). Based on these alignments, several trees are built using RAxML (50) for the ncRNA gene sequences and using neighbour-joining and maximum- likelihood for the flanking sequence. These trees are merged using the treemerge algorithm implemented in TreeBeST. Finally, orthologues and paralogues are extracted in the same way as for protein-coding genes. Detailed methodology for the ncRNA analysis is provided in a companion paper (45).

### Ensembl Protein Families

While GeneTrees are used to infer orthologues and paralogues, the Ensembl Protein Families provide links between the proteins annotated in Ensembl and UniProtKB based on sequence similarity (http://www.ensembl.org/info/genome/compara/family.html; Figure 4C). Ensembl Protein Families are built using all proteins from all the species in Ensembl, supplemented with all the metazoan sequences from UniProtKB (51). As with the GeneTrees, protein similarities are detected using BLASTP (36). However, we use a Markov Clustering algorithm (MCL) (52) to define the families based on the protein similarity scores. Finally, all the sequences in each family are aligned using MAFFT (39).

Each family is named using the description of the member proteins. We require at least 40% of the proteins with an informative description match the consensus description or the family is described as ‘AMBIGUOUS’. The percentage of proteins with an informative description (or part of it) that matches the family name is defined as the score for that family name. If the score is 0, the family name is set to ‘UNKNOWN’.

Ensembl Release 80 has 1 118 000 families, but ∼65% of them represent orphan UniProtKB entries. Compared with GeneTrees, the Ensembl Protein Families are more stringent as only a few Ensembl Protein Families span more than one GeneTree (<2%). On the other hand, ∼80% of the GeneTrees correspond to more than one Ensembl Protein Family. In other words, the families usually represent subsets of the GeneTrees and contain highly similar proteins. More distant relationships will be detected in the GeneTrees only.

### Stable identifiers

Stable identifiers, or stable IDs, are names that can be tracked across Ensembl releases. Stable IDs are provided for both GeneTrees and Ensembl Protein Families and, in both cases, the assignment of a stable ID is based exclusively on the content of proteins in a given tree or family. Neither the topology of the tree nor the orthologues extracted from it are assigned stable IDs.

To assign stable IDs for a new Ensembl release, the proteins present in the new and previous release are compared. If the set of shared proteins in a cluster is exactly the same between releases, the stable ID is kept. If not, the situation may represent complex splits and merges among groups, which is resolved using a greedy algorithm that favours keeping the same stable IDs for larger families. Each stable ID includes a version number, which is increased every time the stable ID is assigned via non-perfect matching. For ncRNA trees, the Rfam ID of each family acts as a stable identifier.

For the Ensembl Protein Families, typically >90% of the clusters are matched perfectly from one release to the next and only a few are matched inexactly. In the case of the GeneTrees, ∼80–90% of the trees are matched perfectly and another 5–10% are usually matched incompletely.

### Quality control

We employ three levels of Quality Control (QC). Level one consists of checks within our production pipelines that test for systematic errors. Some of these check for inconsistencies within the data, such as unexpectedly large or small numbers of results or substantial differences between the current and previous Ensembl releases. Level two QC is a series of data integrity tests (called ‘health checks’ in our documentation) performed directly on the underlying database, using Java as an orthogonal method to our Perl API, to detect data errors that could be masked by undiscovered errors in the API code. This level of QC confirms that data in the Ensembl Compara and Core databases (53) are consistent with each other, and runs regression tests for errors that have occurred in previous Ensembl releases. The final level of QC starts with an Ensembl Compara database release candidate and consists of a test Ensembl web server and manual inspection of select pages and data. This level confirms that any new data tracks are visible, that any new visualization features are working, and that all other data are displayed as expected.

## Data visualization and access

We provide a variety of methods for data visualization and data access specifically designed to maximize the value of the comparative genomics data described above in the context of the rich genome annotation available within Ensembl. The visualization on all of the pages described below can be customized via the ‘Configure this page’ link on the left hand side of each page.

### Comparative tracks in location view

**Figure 6.**
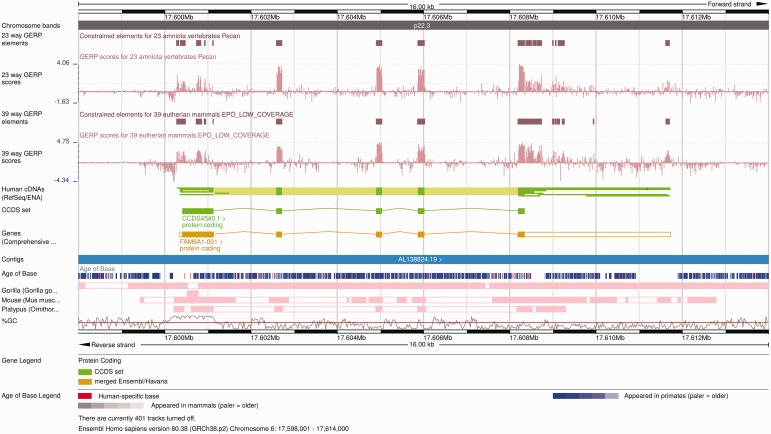
Alignment and conservation tracks on the Location view. The image shows the 23-way amniote and 39-way eutherian conservation scores (pink wiggle tracks) and the corresponding constrained elements (brown blocks) on the FAM8A1 locus. The dark pink tracks at the bottom show the pairwise alignments of this region to the gorilla, the mouse and the platypus genomes. Each element represents an aligned block. These are connected in so-called nets that represent a series of alignment blocks in a congruent order and orientation. There is a secondary block in the gorilla pairwise alignment track, in the centre of the first FAM8A1 exon that represents a break in the continuity between human and gorilla in this region. Finally, the Age of Base track is displayed just below the contig line, and shows the how old each base of the genome is, ranging from human-specific mutations (in red) to primate-wide (shades of blue) and mammal-wide (shades of grey).

The Ensembl Location view shows a region of interest in a given genome with gene annotations and other features shown as horizontal tracks. Genomic alignments can also be displayed on the Ensembl Location view as individual tracks (Figure 6). Pairwise alignments can be displayed in compact mode, showing only the location of these alignments on the genome of interest, or in normal mode, which includes the structure of the alignment nets. Selecting one alignment opens a pop-up menu with more information on both that alignment block and the whole net. Links to additional views are available on the same pop-up menu, including specific options to view the alignment in text or graphical mode.

Multiple alignment tracks show the region in the genome of interest included in the alignment. Selecting a multiple alignment feature provides the coordinates of the alignment on the current genome and links to the alignment views described in the following section. Due to the amount of data, the pop-up menu cannot display all of the regions from the other species included in the alignment.

An important summary analysis based on multiple alignments is the detection of conserved regions in the genomes. Per-base conservation scores are shown as wiggle plots, a continuous data representation where tall bars represent highly conserved bases. The conservation scores are stored at different resolutions to support various levels of genomic context in a single view. The number of pixels in the image and the length of the region to display are used to determine the ideal resolution level for each view. In addition to the per-base conservation scores, constrained elements can be displayed on a separate track. The information shown when selecting a constrained element includes the location of the element on all species, plus the score and *P*-value of the element itself.

Finally, the Age of Base track summarizes the ancestral sequences predicted in the placental EPO alignment (Figure 6). Each position of the human genome is colored according the oldest taxon that possesses the same base at that position. An average region normally exhibits shades of blue, which indicate changes in the primate lineage.

### Alignment views

**Figure 7.**
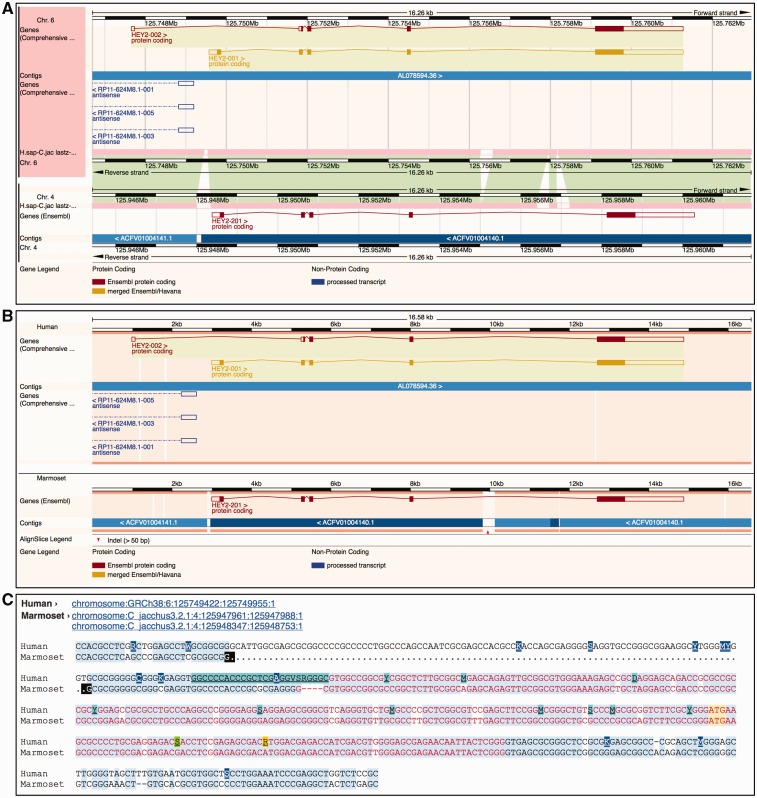
Different alignment views in Ensembl. (**A**) Region Comparison view for the human and marmoset HEY2 genes. The top part of the panel shows the human locus while the bottom half represents the marmoset locus. As in the Location view (Figure 6), the dark pink tracks show the pairwise alignments. The green areas link each part of the alignment blocks, showing the connections between both genomes. (**B**) The graphic alignment view for the same region. The human and marmoset sequences are stretched to accommodate the alignment gaps. The vertical white segments in the background color show these gaps. The marmoset sequence is made of several fragments, as indicated by the alignment. (**C**) Base-pair detail of the alignment for the first exon. Exonic sequence is highlighted in red, start ATG codons in yellow and sequence variants are coded in different colors. At the top of the alignment, the user is presented with the list of loci in this alignment. The marmoset sequence is split in two different segments. The black marks highlight the edges of the aligned regions.

Ensembl provides three different ways to visually represent whole-genome alignments (Figure 7). In Region Comparison view two or more species can be viewed side-by-side with annotation features completely shown in their original genome coordinate system. The two other displays focus on the alignment itself, either in graphical mode or in text mode. The graphical mode provides an overview of how genomic features such as genes align to one another across species, while the text mode shows the details of how the nucleotides are aligned.

The Region Comparison view (Figure 7A) shows genomic regions of different species stacked relative to each other. The alignments between these species are shown as lines between the individual windows, with each line connecting homologous positions between the two regions. This view is especially well suited for highlighting insertions, deletions and small-scale inversions between any two genomes.

The graphical Alignments (image) view (Figure 7B) uses all the alignments in the region of interest to display the sequences and genomic features from the different species on a common coordinate system defined by the alignment rather than accommodating the coordinate systems from the aligned genomes as is displayed in the Region Comparison view. In other words, on each alignment block, homologous positions from the individual genomes sequences are aligned vertically on the display by padding the displayed regions if necessary. As a result, it is trivial to compare the location, boundaries and structure of the features among the different species.

The text-based Alignments (text) view (Figure 7C) focuses on the alignment at the base pair level. It complements the graphical display and is better suited for smaller genomic regions. Several layers of information can be shown on the sequence. For instance, exons, start and stop codons and SNPs can be switched on or off using the configuration panel. Different font coloring and highlighting schemes mark these features in the view. It is also possible to highlight conserved positions in the alignment, which are calculated on the fly where the majority of the sequences agree with each other. This approach does not use the conservation data obtained from the multiple alignments enabling us to highlight the agreement and disagreement in any alignment, including pairwise alignments.

### Synteny view

**Figure 8.**
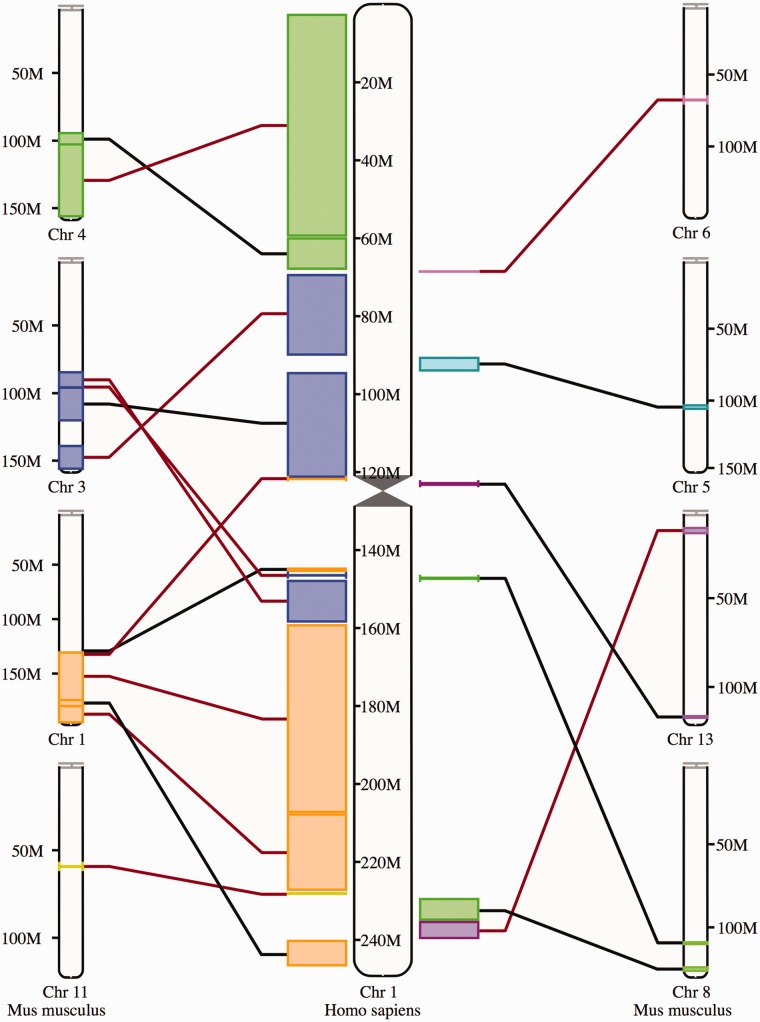
Synteny view. The view shows the syntenic blocks between human chromosome 1 and the mouse chromosomes 1, 3, 4, 5, 6, 8, 11 and 13. The blocks are linked between the human and the mouse with a black line if they appear in the orientation and with a red line if they are inverted in one species with respect to the other.

The synteny display is a chromosome-level comparison between two species, which provides a visualization of the large-scale chromosome rearrangements between the species. As such, it is only available for species that have their genome assemblies anchored to chromosomes. The syntenic blocks are drawn in different colors according to the matching chromosome in the other species (Figure 8). Blocks inverted in a genome with respect to the other are shown with a red line. In addition to this specific view, syntenic regions can also be shown as a track on Location view.

### Orthologous and paralogous

Orthologous and paralogous genes are displayed in Ensembl as tables with dynamic filter functionality. The similarity between each pair of genes relates to the sequence identity in the protein alignment used for the GeneTree reconstructions. The tables provide details such as gene locations and links to view the alignment between the genes or to a Region Comparison display with the homologous genes side-by-side.

For paralogues, we include an estimation of when the paralogues diverged using the taxonomic information from the GeneTrees. For orthologues, we provide the pairwise *dN*/*dS* value. We also provide a summary table with the type and number of orthologues in each clade to help effectively manage the increasing number of species in Ensembl.

### GeneTree view

Phylogenetic trees are displayed on the GeneTree view. As shown in Figure 5, the view is split in to two parts. The left panel shows the tree itself while the right panel shows a summary overview of the corresponding multiple alignment. Duplication nodes are highlighted in red, ambiguous nodes (12) are shown in cyan and gene split events are colored in light brown. Branches longer than one substitution per site are scaled down one or two orders of magnitude as appropriate, so that the topology of the tree is easily readable. The multiple alignment panel gives an overview of the gaps in the alignment as well as the exon boundaries, which are displayed with tick marks.

When navigating from the Ensembl Gene view page, the gene of interest is highlighted in red; its within-species paralogues are displayed in blue. Navigation from the orthologues table to the GeneTree view results in highlighting the pair of orthologous genes and their within-species paralogues. By default, the tree is colored according to the taxonomy of the species.

Nodes of the tree can be collapsed or expanded by selecting them. Several options exist for auto-collapsing nodes based on the topology of the tree, for example collapsing all duplication nodes. It is also possible to hide genes from pre-defined clades, such as rodents, or from low-coverage genomes. The configuration panel allows the users to change these and other options and set new defaults to suit their preferences. For example, options exist to collapse nodes, specify whether exon boundaries will be shown, and to define the default coloring mode (foreground, background or none).

Additional information on any gene or any internal node is available by selecting it. The resulting pop-up menu provides links to other Ensembl views; to external resources such as TreeFam (20), PhylomeDB (17) and Genomicus (54); and to Jalview (55), a Java-based alignment and tree editor.

From any internal node of the tree, it is possible to export the underlying alignment and the subtree in both multi-FASTA and New Hampshire format. When the resulting sub-tree contains a relatively few genes (up to 10), it is also possible to display these genes in the Region Comparison view described earlier.

### Gene gain/loss view

The Gene gain/loss tree view maps the number of copies of each gene in each species in a given GeneTree. This information is shown on a taxonomic tree, where internal nodes display the total number of ancestral copies as estimated by CAFE (42). In other words, for any selected gene in any species, the Gene gain/loss tree view provides both the number of extant homologues as well as an estimate of the number of homologues at each ancestral node. Branches leading to major expansions or contractions are highlighted in red or green, respectively. Selecting a node opens a pop-up menu with additional information on the CAFE results for that particular taxon. Not all trees include genes from all the species: the configuration panel supports switching between displaying the full species tree or a reduced version that ignores ancestral species not represented in this tree.

### Ensembl Protein Families

Information about the Ensembl Protein Families is linked from the left side of the Gene view page and is provided as table with a list of all the Ensembl Protein Families in which the specific gene is found. For each Ensembl Protein Family, several detailed views exist including a full list of proteins from all Ensembl species in that family as well as other genes from the same species in that family. The latter view shows the karyotype of the genome, if available, with the location of all the family members highlighted. Lastly, either the entire alignment or the alignment of the Ensembl proteins only can be exported to JalView for additional visualization options.

### Alternative access to the data

In addition to the web interface, the comparative genomics data in Ensembl are accessible through our public MySQL database server (ensembldb.ensembl.org), via direct download from our FTP site (ftp://ftp.ensembl.org) or using our Perl API (http://www.ensembl.org/info/docs/api/compara/index.html), which includes a variety of example scripts for downloading complex datasets. Alternative programmatic access to some data is also available through our REST API (http://rest.ensembl.org) (56).

The raw MySQL tables are available on our FTP server to support installation of local copies. The FTP server also includes flat files for some of our data, including the whole-genome multiple alignments and the gene trees as EMF (Ensembl Multi Format) files. EMF has been designed especially to provide per-position alignment scores. Sequences are represented in columns and homologous positions in rows. Sequence conservation scores are included with the alignments in an additional column containing these values. Gene trees and homologies are also available in XML formats (OrthoXML and PhyloXML).

## Discussion

The Ensembl comparative genomics infrastructure has been developed for the analysis of the chordate genomes present in Ensembl although it has been successfully used for other clades such as plants (25) and bacteria (24). Ensembl’s resources are largely complementary to those provided by other resources. For instance, OMA offers orthology predictions for a much broader set of species (1850 in the 18th release) (21). It also offers OMA stand-alone, which is designed for the analysis on any set of genomes in a local environment. Panther also infers phylogenetic trees on a large set of species with the specific aim of facilitating high-throughput annotation of genes (19). PhylomeDB collects both multiple alignments and phylogenetic trees in so-called phylomes (17). Each phylome represents the set of phylogenetic trees for all the genes of a given species and they are provided for a broad variety of species including human, plants, prokaryotes and yeast.

There are relatively few sources for chordate whole- genome multiple alignments. These include VISTA, which currently distributes a 5-way primate multiple alignment and the GenomeVISTA toolkit (57) and the UCSC Genome Browser with a variety of MultiZ alignments (58). Multiple alignments from UCSC are not synchronized across species however: the human GRCh37 assembly (hg19) genome browser includes a 100 species multiple alignment, which includes the mouse GRCm38 (mm10) assembly, but the corresponding mouse genome browser provides only a 60-way multiple alignment leading to non-compatible conservation tracks for these species (14). The UCSC Genome Browser also provides PhyloP conservation scores for these multiple alignments (59).

### Scalability

The Ensembl Compara database provides pre-calculated alignments, gene trees, orthology predictions, syntenies, conservation data and other information for almost 70 species. In total, these data require ∼5 million CPU hours to create. To facilitate updates between Ensembl’s regular releases, our workflows reuse data from one release to the other. A typical release requires only about half-a-million CPU hours, while releases featuring updated assemblies for the human, mouse or zebrafish genomes require more due to the substantial number of pairwise alignments with these species. The most expensive calculations are the all-vs-all pairwise BLAST alignments used in the GeneTree, Ensembl Protein Family and Mercator pipelines. For maximal efficiency, we have implemented a mechanism to reuse the BLAST results whenever possible.

Portions of the Ensembl Compara database grow quadratically with the number of species and for Ensembl release 80, required about 320 GB of disk space for the data and the indexes. If the current trend continues, the database will be ∼800 GB in size for 100 species.

### Documentation

In the Documentation section of the Ensembl website, we include information about the different data types and technical information on our analysis pipelines. We also provide summary statistics for all the pairwise alignments. There are useful examples, videos and tutorials freely accessible from the website. New features are publicized on the Ensembl blog (http://www.ensembl.info). Specific questions can be directed to the Ensembl helpdesk (helpdesk@ensembl.org).

### Conclusions

Comparative genomics analyses are vital for many genomics-based research studies and are a central part of the genome resources provided by Ensembl. Our most comprehensive resources and the majority of our usage are concentrated on the human, mouse, rat, chicken and zebrafish genomes, with other species often used for comparative and evolutionary analyses only. The Ensembl comparative genomics infrastructure, which supports all of these uses, is designed to be species-independent: it allows us to store one single copy of the alignments, trees, and orthologues, and make them accessible for all the species in Ensembl. This aspect of the Ensembl Compara database enables full consistency of all of our comparative genomics resources with every release. This unique and powerful feature of Ensembl ensures that the same alignments are presented in all situations for all species.

Ensembl’s visualization options present data over a wide range from whole karyotype synteny to individual aligned base pairs. These data resources and visualization options provide numerous ways for data to be explored and incorporated directly into a variety of analyses, as well as to help understand newly sequenced genomes and to aid the interpretation of genomic data or features from an evolutionary point of view. Indeed, the Ensembl comparative genomics resources have contributed directly to the analysis and interpretation of several genome sequencing projects including the orang-utan (60), gorilla (61), tammar wallaby (62), chicken (63), turkey (64), zebra finch (65) and lamprey (66). Our comparative data have also played a role in the analysis efforts of ENCODE (67) and the 1000 Genomes Project (68). These efforts have helped shape our resources through direct participation in key use cases.
